# Generative AI in Medicine and Healthcare: A Comprehensive Review of Foundational Technologies, Clinical Applications, and Future Perspectives

**DOI:** 10.14789/ejmj.JMJ25-0036-R

**Published:** 2026-04-10

**Authors:** TOMOSHIGE NAKAMURA, WATARU UCHIDA, AKIRA YAMAMOTO, SHIGEKI AOKI

**Affiliations:** 1 Faculty of Health Data Science, Juntendo University, Chiba, Japan; 1 Faculty of Health Data Science, Juntendo University, Chiba, Japan; 2 Graduate School of Medicine, Juntendo University, Tokyo, Japan; 2 Graduate School of Medicine, Juntendo University, Tokyo, Japan

**Keywords:** generative artificial intelligence, large language models, medical imaging, digital health, clinical workflow

## Abstract

Generative Artificial Intelligence (AI) is poised to induce a paradigm shift in medicine and healthcare. This review provides a comprehensive overview of its foundational technologies, clinical applications, and pathways to practical integration. We first explain the principles of three core technologies: Transformers, exemplified by Large Language Models (LLMs); Diffusion Models for high-fidelity data generation; and Neural Radiance Fields (NeRF) and 3D Gaussian Splatting (3DGS) for 3D scene synthesis. We then systematically review their applications across diverse domains: automating clinical documentation, accelerating drug discovery, enhancing medical imaging diagnostics, and innovating surgical simulation. To bridge the gap to real-world implementation, we address critical system-level challenges, discussing practical solutions such as Retrieval-Augmented Generation (RAG) to mitigate hallucinations, on-premises LLMs to ensure data security, and no-code platforms to empower clinician-led development. Finally, we examine critical ethical, legal, and social issues―including data bias, interpretability, and accountability―emphasizing the need for a robust governance framework. This review underscores that generative AI is evolving beyond a mere efficiency tool into a powerful partner capable of augmenting the expertise of healthcare professionals and fundamentally shaping the future of medicine.

## Introduction

Recent advances in Artificial Intelligence (AI) are reshaping the landscape of medicine and healthcare. A symbolic milestone was the awarding of the 2024 Nobel Prizes in Physics and Chemistry for foundational work on artificial neural networks and AI-powered protein structure prediction, respectively. This event underscores that AI has become a foundational technology, enabling a new paradigm of data-driven scientific discovery that complements traditional hypothesis-driven science^1)^. At the heart of this technological wave is Generative Artificial Intelligence, a class of models with the capacity to create a wide variety of data, including text, images, and molecular structures.

Meanwhile, the modern healthcare sector faces unprecedented challenges. A particularly pressing issue is the escalating administrative burden in clinical practice, where physicians and medical staff reportedly spend a substantial portion of their time on documentation tasks—such as entering data into electronic health records (EHRs) and drafting reports—rather than on direct patient care^2)^. This administrative overhead not only compromises the quality of care but also contributes to clinician burnout, thereby posing a significant obstacle to a sustainable healthcare system.

Against this backdrop, generative AI is emerging as a promising solution to address clinical inefficiencies and enhance the quality of care. Large Language Models (LLMs), built upon the attention mechanism^3)^, exhibit an advanced capacity for natural language understanding and generation, and thus have the potential to dramatically improve the efficiency of automated medical documentation and summarization. Similarly, Diffusion Models^4)^, which operate by reconstructing high-fidelity data from noise, are being applied to tasks ranging from medical image super-resolution to the prediction of complex biomolecular interactions, as exemplified by AlphaFold3^1)^. Their applications are expanding across the healthcare spectrum, from diagnostic support to drug discovery. Furthermore, technologies such as Neural Radiance Fields (NeRF)^5)^ and 3D Gaussian Splatting (3DGS)^6)^, which synthesize novel 3D views from 2D images, are poised to transform surgical simulation and medical education.

The purpose of this review is to provide a comprehensive overview of the applications of generative AI in medicine and healthcare from three distinct perspectives. First, we outline the principles of three foundational technologies: the Transformer, the Diffusion Model, and Neural Radiance Fields (NeRF) and 3D Gaussian Splatting (3DGS). Second, we systematically review their applications across diverse domains—including clinical workflow optimization, drug discovery, medical imaging, and medical education—drawing upon the latest research findings. Third, we examine system-level approaches for practical implementation, such as Retrieval- Augmented Generation (RAG)^7)^ and on-premises model deployment, and discuss the associated challenges of data security, ethics, and governance.

Through this review, we aim to demonstrate that generative AI is more than an efficiency tool; it is poised to become a powerful partner that can augment the expertise and clinical judgment of healthcare professionals. The remainder of this paper is organized as follows. Section 2 provides an overview of the core technologies. Section 3 discusses their application to clinical challenges through specific use cases. Section 4 addresses the practical transition of these technologies into real-world settings. Section 5 examines the broader challenges associated with implementation. Finally, Section 6 synthesizes these discussions to offer a perspective on the future of generative AI in medicine and healthcare.

### Literature review methodology

To ensure a comprehensive review, we conducted a systematic literature search covering the period from January 2017 to May 2025. We queried databases including PubMed, IEEE Xplore, Google Scholar, and arXiv to encompass both peer-reviewed clinical studies and the latest algorithmic advancements in computer science. Search queries combined keywords such as “Generative AI,” “Large Language Models (LLMs),” “Transformer,” “Diffusion Models,” “Neural Radiance Fields,” “3D Gaussian Splatting,” “Medical Imaging,” “Clinical Documentation,” and “Drug Discovery.” We prioritized peer-reviewed articles and high-impact preprints or conference papers that explicitly address the technical foundations and clinical applications of these technologies. Exclusion criteria included non-English publications and articles lacking technical or clinical validation, with the exception of specific Japanese regulatory guidelines essential for the discussion on governance frameworks.

### Foundational technologies of generative AI

Recent progress in generative AI is largely attributable to several architectural breakthroughs in neural networks. This section outlines the operating principles of three foundational technologies for medical and healthcare applications: the Transformer, the Diffusion Model, and Neural Radiance Fields (NeRF) and 3D Gaussian Splatting (3DGS).

### Transformer and large language models

The field of Natural Language Processing (NLP) advanced significantly with the introduction of the Transformer architecture^3)^. This architecture overcame a key limitation of its predecessors—such as Recurrent Neural Networks (RNNs) and Long Short-Term Memory (LSTM) networks—which struggled to capture long-range dependencies in sequential data. The Transformer achieved this improvement through a mechanism called Multi-head Attention, which is composed of multiple Self- Attention heads.

The core principle of Self-Attention is its capacity to dynamically weigh the importance of each element (e.g., a word) relative to all other elements in a sequence. Based on these weights, it updates the representation of each element. This process is analogous to a clinician's cognitive workflow when reading a medical chart: to interpret a specific finding (e.g., fever), the clinician unconsciously focuses on related information (e.g., chills or an elevated white blood cell count) while directing attention away from irrelevant details (e.g., a recent trip or a fall). This mechanism allows the Transformer to acquire a dynamic, context-aware representation for each word. By arranging multiple Self-Attention layers in parallel (Multi-head Attention), the model can simultaneously extract diverse aspects of the text, such as grammatical structure and semantic relationships.

A Large Language Model (LLM) is a Transformer model that has acquired sophisticated language capabilities through self-supervised learning (e.g., predicting the next word in a sentence) on vast amounts of text data from the internet. Furthermore, fine-tuning techniques such as Reinforcement Learning from Human Feedback (RLHF)^8)^ are employed to align the model's output with human intent, thereby reducing the risk of generating harmful or factually incorrect responses. These technological advancements form the foundation of the conversational and text-generation abilities of modern LLMs.

### Diffusion model

Diffusion Model^4)^ is a generative approach particularly effective for high-quality image synthesis. It operates in two complementary phases—a forward diffusion phase and a reverse denoising phase—through which the model learns to reconstruct realistic data from noise.

(i)**Forward process**: A predefined schedule gradually adds small amounts of Gaussian noise to the original data (e.g., an image) over many steps until it becomes almost indistinguishable from random noise. This provides a stable training target and helps the model cover a wide range of possible variations in the data.

(ii)**Reverse process**: Starting from the noise-like state, a neural network (typically a U-Net) is trained to predict and remove the noise step by step, effectively reversing the forward process. Repeating this denoising procedure yields new, realistic samples that follow the distribution of the training data. Training focuses on learning this reverse-time denoising behavior.

This stepwise denoising mechanism enables diffusion models to achieve high fidelity and diversity compared to earlier generative methods. They also offer robust and stable training characteristics that are valuable in medical imaging workflows. Furthermore, Latent Diffusion model^9)^ execute both diffusion and denoising in a lower-dimensional latent space rather than pixel space, which reduces computation and memory costs while preserving image quality after decoding.

Diffusion models naturally support conditional generation, in which the reverse process is guided by external inputs—for example, text prompts or reference images—so that outputs align with user intent^10)^. In practice, this guidance can be tuned to balance faithfulness to the prompt and visual realism, enabling applications such as super-resolution, denoising, inpainting, and cross-modal synthesis under appropriate clinical validation.

### 3D scene representation

Significant progress has been made in techniques for representing 3D scenes and synthesizing novel views from a collection of 2D images taken from multiple viewpoints. The pioneering technology in this domain is Neural Radiance Fields (NeRF)^5)^ which represents a 3D scene as a continuous neural function. This function, given a 3D location and a viewing direction, outputs the expected color and volume density at that point. Trained on multi-view images with known camera poses, the network implicitly learns the scene’s geometry and appearance. Once trained, novel viewpoints can be rendered by casting rays through the scene and accumulating the predicted color and density along each ray, producing photorealistic images from arbitrary camera positions. This implicit representation yields high fidelity but typically entails long training and rendering times.

To address these efficiency limits, 3D Gaussian Splatting (3DGS)^6)^ models the scene explicitly as a large set of 3D Gaussian primitives, each with position, shape, orientation, color, and opacity. These primitives are then rasterized efficiently on the GPU, enabling real-time interaction while maintaining high visual quality. In practice, 3DGS offers a substantial speed-up in both training and rendering compared to NeRF, making it well-suited for interactive applications such as virtual walkthroughs and simulations.

From a medical perspective, these technologies provide a foundation for immersive visualization—for example, reconstructing operative fields from endoscopic or intraoperative videos, or creating anatomy-aware learning content from routine imaging. While Section 3 discusses concrete applications and constraints, it is worth noting here that data capture conditions (e.g., limited viewpoints, motion, or tissue deformation) strongly influence reconstruction stability and performance; thus, workflow-appropriate data collection and validation are essential for successful deployment in healthcare settings.

## Generative AI applications in medicine and healthcare

The foundational technologies of generative AI, as outlined in Section 2, are being applied to address diverse challenges across the medical and health sectors. This Section categorizes these applications into four key domains, reviewing the current state and future potential by drawing upon the latest research.

### Automation of clinical workflow and documentation

One of the most significant challenges in clinical practice is the administrative burden associated with medical documentation. Large Language Models (LLMs), based on the Transformer architecture, are emerging as a powerful solution to this problem. Applications are being developed to perform real-time transcription of doctor-patient conversations, structure the content into summaries like EHR progress notes (SOAP format), and automatically generate discharge summaries^11)^. Indeed, efforts to generate high-quality discharge summaries from fragmented EHR information using LLMs are underway, along with the development of datasets to validate their practical utility^12, 13)^. Furthermore, a study investigating the impact of Ambient Scribing Technology—an AI tool that automatically listens to clinical conversations and drafts chart notes—suggested that it significantly reduces the time clinicians spend on documentation, decreases after-hours work, and alleviates their perceived psychological burden^14)^. The evaluation of techniques for automatically assigning medical codes from clinical records using LLMs is also an active area of research^15)^. These advancements are expected to contribute to the overall efficiency of outpatient care processes, leading to developments such as automated patient questionnaire generation from patient-entered symptoms and decision support systems for medical billing.

However, rigorous evaluation is essential for deployment. A study on Med-PaLM, a large language model developed by Google^2)^, verified its ability to encode specialized medical knowledge, generating answers comparable to those of clinicians. At the same time, the study highlighted the potential for inappropriate or inaccurate information, underscoring that the generation of non-factual content (i.e., hallucinations) remains a significant challenge. Consequently, a verification and approval process for LLM-generated text is necessary, making a human-in-the-loop operational model indispensable.

**Figure 1:**
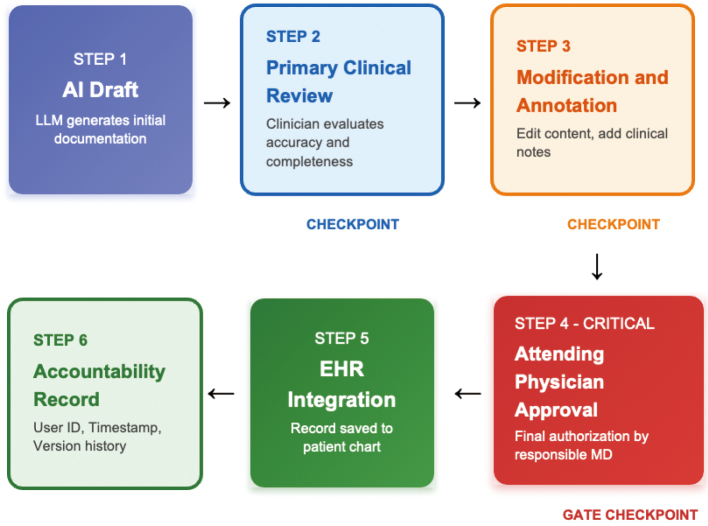
A human-in-the-loop approval workflow for AI-generated documentation This flowchart delineates the critical checkpoints for ensuring clinical accuracy and accountability. The process mandates physician intervention for modification (Step 3) and final approval (Step 4) to mitigate automation bias, followed by secure EHR integration and immutable logging (Steps 5-6).

Figure 1 illustrates a robust human-in-the-loop verification framework designed to ensure patient safety and accountability. The workflow emphasizes active clinician engagement at multiple checkpoints. Specifically, Step 2 (Primary Clinical Review) requires the clinician to verify that the AI has not misheard or mistranscribed the dialogue and to correct any inaccurate expressions. Subsequently, Step 3 (Modification and Annotation) involves the manual addition of nuances or physical findings that the AI might have overlooked or captured insufficiently. These intermediate interventions are crucial for maintaining the quality of the record.

The final authorization stage, Step 4, serves as the critical gatekeeper but is also susceptible to "automation bias." This phenomenon occurs when the high fluency and coherent structure of AI-generated text lead clinicians to lower their vigilance, causing them to overlook subtle but critical errors—such as laterality (right vs. left) or dosage units (mg vs. g). To mitigate this risk, the user interface for final review must be designed to reduce cognitive load while alerting the physician to potential pitfalls. Effective UI strategies include "clickable evidence," where selecting a generated sentence plays back the corresponding segment of the source audio, and visual highlighting of low-confidence transcriptions. By integrating these safeguards, the system can support efficient documentation while preventing the oversight of hallucinations before the record is finalized in the EHR (Step 5) and logged for accountability (Step 6).

Moreover, a randomized clinical trial that examined whether physician access to ChatGPT Plus improved diagnostic reasoning compared to using conventional diagnostic resources found no statistically significant improvement in diagnostic performance^16)^. This result indicates that merely providing clinicians with access to LLMs is insufficient for clinical use and underscores the need for further technological development, professional training, and a considered approach to the partnership between clinicians and AI.

### Drug discovery and structural biology

The accurate prediction of the three-dimensional structures of biomolecules, such as proteins, is a critical challenge in elucidating biological phenomena and in drug discovery research. In this domain, generative AI is becoming a tool that accelerates the pace the traditional experimental science.

A major milestone was the development of AlphaFold2, which adopted a Transformer-based architecture^17)^. AlphaFold2 successfully predicted the monomeric structure of proteins from amino acid sequence information alone with high accuracy, driving significant progress in structural biology. Its successor, AlphaFold3^1)^, extended the underlying model to a Diffusion Model, enabling the prediction of complex structures and interactions not only of proteins but also of other molecules like DNA, RNA, and ligands. This has made it possible to simulate how drug candidate compounds bind to disease-related target proteins. Consequently, this technology is expected to dramatically accelerate the target identification and lead optimization phases of the drug discovery process.

### Diagnostic imaging

Diagnostic Imaging is one of the most anticipated areas for AI application in medicine. The increasing workload associated with interpreting medical images such as CT and MRI scans has created a demand for AI systems that can provide fast and accurate diagnoses. In this field, the introduction of the Diffusion Model is expected to yield two major advancements.

The first is its application to image quality improvement (super-resolution). For instance, research is progressing on applying Diffusion Models to low-resolution and high-noise images—obtained from low-dose CT to reduce radiation exposure or from high-speed MRI to shorten examination times—to transform them into high-quality images suitable for diagnosis^18)^. While Generative Adversarial Networks (GANs) have been widely explored for these tasks, they face significant hurdles in medical contexts, particularly regarding training instability (e.g., mode collapse) and the risk of generating plausible but clinically non-existent artifacts (hallucinations). In contrast, Diffusion Models offer superior stability and controllability, enabling better preservation of physical properties such as CT Hounsfield Units (HU) or MRI signal-to-noise ratios (SNR). Although they typically require higher computational cost at inference time compared to the single-pass generation of GANs, their ability to produce high-fidelity outputs with fewer artifacts makes them increasingly preferred for diagnostic tasks^10)^. A detailed comparison of these architectures is provided in Table 1.

The second is its application to data augmentation for training on rare diseases. When developing a diagnostic AI for a rare disease where sufficient training data is unavailable, Diffusion Models can be used to generate realistic synthetic images, for example, by synthesizing only the lesion onto images from healthy individuals. This approach is expected to improve the robustness and generalization performance of the AI model. Furthermore, research on multimodal AI that automatically generates radiologist reports from chest X-rays and CT images is actively being pursued, with the potential to reduce the workload of radiologists and prevent diagnostic errors^19, 20)^.

**Table 1:** Comparison of GAN-based vs. diffusion-based generation in medical imaging

Dimension	GAN-based generation	Diffusion-based generation	Practical guidance (medical imaging)
Training stability	Sensitive to hyperparameters; mode collapse risk	Typically more stable optimization	When training resources and tuning capacity are limited, diffusion is often operationally safer
Artifact tendency	Texture-like “hallucinated” details; sharp but potentially non-faithful	Stepwise denoising tends to preserve global structure; artifacts can appear as over-smoothing or residual noise	For diagnosis-adjacent tasks, prioritize faithfulness over perceptual sharpness
Physical consistency (e.g., CT HU, SNR)	Can violate physical intensity statistics if not constrained	Easier to incorporate conditioning/consistency constraints across denoising steps	Add explicit intensity/physics constraints and evaluate HU/SNR distributions, not only visual quality
Data requirement	Can work with moderate data but may overfit; diversity issues	Often benefits from large diverse datasets; can be robust under proper conditioning	For rare disease augmentation, carefully validate distributional shift and bias amplification
Inference speed	Usually fast (single forward pass)	Slower (multi-step sampling); can be accelerated (latent diffusion / fewer steps)	Real-time constraints may favor GAN; offline generation can favor diffusion
Controllability	Conditional GANs possible but can be brittle	Strong conditional generation; flexible guidance	Prefer diffusion for controlled augmentation/inpainting and uncertainty-aware workflows
Clinical evaluation	“Looks realistic” can mislead; needs rigorous downstream validation	Also requires rigorous validation; easier to tie outputs to conditioning evidence	Evaluate with task-level metrics and clinician review; document failure modes

This table summarizes the trade-offs between Generative Adversarial Networks (GANs) and Diffusion Models, focusing on stability, physical consistency, and practical suitability for clinical applications. (HU: Hounsfield Units, SNR: Signal-to-Noise Ratio).

### Medical education and surgical simulation

The acquisition of surgical skills and anatomical knowledge requires a high degree of three-dimensional spatial awareness. In this field, which has traditionally relied on 2D video materials and limited opportunities for anatomical dissection, NeRF and 3DGS are expected to provide more immersive and realistic educational and training environments^21-23)^.

However, technical challenges remain. In applications such as endoscopic surgery, the target objects are constantly deforming, and the endoscopic viewpoint is often monocular and narrow, which can cause Structure-from-Motion (SfM) algorithms used for camera pose estimation to fail^24)^. These conditions necessitate the development of technologies that can achieve stable and fast 3D scene reconstruction. Additionally, practical constraints in the operating room, such as the difficulty of installing dozens of cameras for hygienic reasons, result in sparse and fixed viewpoints^25)^.

## A systems-level approach to the practical implementation of generative AI

Transitioning generative AI from the proof-of-concept stage to real-world clinical environments requires an architecture that addresses not only model performance but also system-level challenges including reliability, data security, and development agility. This Section discusses three system-level approaches for implementation in medical settings: Retrieval-Augmented Generation (RAG), on-premises/local LLMs, and no-code/low-code platforms.

### Ensuring reliability with retrieval-augmented generation

One of the primary challenges of Large Language Models (LLMs) is the phenomenon of "hallucinations"—the generation of information that is not grounded in fact or is inconsistent with the model's training data. For instance, an LLM might cite a non-existent URL as the source for a claim it generates. In a field like medicine, where informational accuracy is directly linked to patient safety, this problem poses an unacceptable risk. A leading solution to this challenge is Retrieval-Augmented Generation (RAG)^7)^.

RAG is a framework that supplements the broad, general knowledge of an LLM with a reliable, task- specific external knowledge source. For example, when a clinician inputs a prompt about a specific patient, the system first retrieves relevant documents from a trusted database, such as internal clinical guidelines, EHRs, or reputable medical literature. This retrieved information is then provided to the LLM along with the original prompt to generate a response. This process constrains the LLM to provide answers based on the timely and contextually relevant evidence rather than relying solely on its static, pre-trained knowledge, thereby mitigating the risk of hallucinations. A practical application of this is TrialGPT^26)^, a system designed to rapidly and accurately match patients to suitable clinical trials. TrialGPT uses an LLM to process patient records, search a database of clinical trials from past cohort studies, and determine a patient's eligibility, explaining the basis for its conclusions. This approach provides the significant advantages of enhancing the reliability of the output and streamlining the verification process.

**Figure 2:**
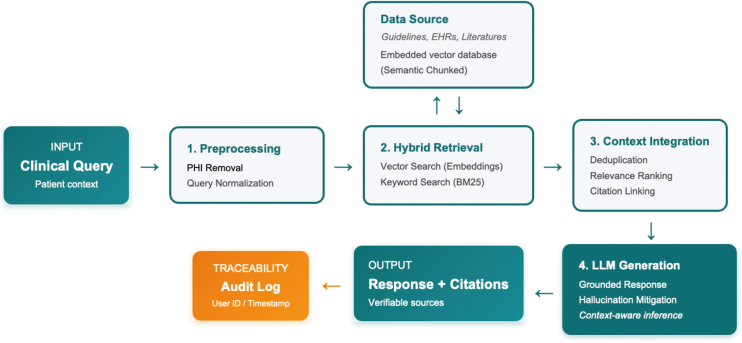
The workflow of a retrieval-augmented generation (RAG) pipeline in healthcare This diagram illustrates the end-to-end process for mitigating hallucinations and ensuring reliability. The workflow integrates privacy protection (PHI removal), hybrid retrieval from trusted sources, and citation-backed generation, underpinned by a comprehensive audit log for traceability and governance.

Figure 2 illustrates a robust RAG pipeline designed specifically for medical applications. As depicted, deploying RAG in clinical settings requires strict operational constraints to ensure transparency and accountability. The workflow begins with the input of a clinical query, which often contains complex patient context. To ensure privacy and data consistency, the query undergoes preprocessing, specifically the removal of Protected Health Information (PHI) and normalization to resolve terminological inconsistencies. Next, the system executes a hybrid retrieval process to fetch relevant documents from a trusted knowledge base—constructed from internal protocols and validated literature. In the generation phase, the LLM is constrained to prioritize this retrieved context over its pre-trained parametric knowledge. Crucially, the model is engineered to append citations to every claim, allowing for immediate verification of the source material. Finally, adhering to the human-in-the-loop principle, the output undergoes clinical review before any action is taken. To further mitigate risks and enable continuous system improvement, maintaining comprehensive audit logs is essential. An effective audit log must ensure full reproducibility of the inference process; this includes recording the anonymized input query, retrieval results, system prompts, the specific context chunks used, the generated output with citations, and the final clinician's decision. Such detailed logging allows for retrospective analysis in the event of an error. Furthermore, the efficacy of this pipeline depends heavily on the rigorous construction of the underlying knowledge base. This involves careful data source selection, semantic chunking strategies, and domain-specific vectorization to capture medical nuances. Optimizing the retrieval strategy (e.g., balancing keyword search with semantic embeddings) is equally critical to ensure the most relevant evidence is surfaced for the LLM.

### On-premises and local LLMs for data security

Protecting patient privacy and ensuring data security are paramount in any medical information system. Most publicly available, cloud-based generative AI services require patient data to be sent to external servers, which may conflict with the security policies of many healthcare institutions and violate regulations such as HIPAA.

One effective approach to overcome this challenge is to build and operate LLMs in an on-premises environment, contained entirely within the healthcare organization's secure network. While operating high-performance LLMs once required vast computational resources, the recent proliferation of open-source models has changed the landscape. High-performance, open-weight models that are commercially viable and can run on relatively modest computational resources are now available (with representative examples including models from the gpt-oss-120B, Llama 3 family, DeepSeek, and Gemma). This trend has made the deployment of local LLMs—whereby an institution hosts model on its own servers and utilize generative AI without transmitting sensitive information externally—a viable option. Cases of Japanese hospitals deploying LLMs on internal servers to assist with medical documentation have already been reported. Table 2 provides a systematic comparison of these deployment models, outlining the trade-offs in security, cost, and governance to support institutional decision-making.

However, deploying these models locally presents unique infrastructure challenges. In practice, on-premises deployment in hospitals is often constrained by limited GPU availability, strict network segmentation, and operational requirements for high availability and auditable access control. These constraints motivate engineering choices such as (i) selecting smaller open-weight models and applying quantization to reduce memory footprint, (ii) limiting context length while relying on retrieval for long-context needs, (iii) implementing request throttling and caching for predictable latency, and (iv) designing comprehensive audit logs (user identity, timestamps, retrieved sources, prompts, and outputs) to support clinical governance.

**Table 2:** Comparison of cloud-based vs. on-premises LLM deployment in healthcare settings

Requirement	Cloud-based LLM	On-premises/local LLM	Practical decision guidance
Security boundary	Vendor-controlled boundary; data transit over external networks	Institution-controlled boundary within local security perimeter	Prefer on-premises when strict data residency, network segmentation, or internal security controls are required
PII/PHI handling policy	Depends on vendor policy and contract terms (retention, logging, training use, access)	Institution can enforce local policies (retention, access, and secondary use)	Choose based on enforceability and auditability of data-handling terms
SLA/availability	Typically supported by vendor-managed SLAs	Depends on institutional operations (redundancy, maintenance, incident response)	Cloud may be advantageous if the institution lacks 24/7 operational capability; on-prem requires robust ops maturity
Audit logs/traceability	Logging scope and accessibility depend on vendor features	Full-stack logging and governance design are possible locally	Healthcare deployments often require detailed audit trails; on-prem enables finer-grained traceability (users, prompts, retrieved sources, outputs)
Latency	Network-dependent; may vary with connectivity and region	Potentially lower and more predictable within local infrastructure	For time-critical clinical workflows, prioritize predictable low latency; hybrid setups can be considered
Operational cost	Often variable (usage-based) plus integration/security overhead	Mostly fixed (hardware procurement, staffing, upgrades) plus electricity/space	Optimize total cost of ownership (TCO) based on utilization profile, peak demand, and staffing constraints
Model update control	Vendor-controlled update cadence; limited control over versioning in some cases	Institution can control versioning, validation, and rollout timing	When regulatory validation and change management are required, tighter update control favors on-prem
Customization	Sometimes constrained (fine-tuning, plugins, tool access, data connectors)	Local fine-tuning, RAG optimization, and domain adaptation are typically easier	If strong local customization is needed (institutional documents, templates, policies), on-prem is often preferable
Compliance & governance burden	Shared responsibility with vendor; requires careful vendor risk management	Institution bears more direct responsibility for compliance and governance	Cloud can reduce infrastructure burden but increases third-party risk management; on-prem shifts burden to internal governance
Data integration	Often requires secure gateways and strict access controls	Direct integration within local EHR/clinical systems may be simpler	Prefer architectures that minimize data movement and support least-privilege access

This table outlines key considerations across security, governance, and infrastructure domains to guide institutional decision-making. (SLA: Service Level Agreement, PII: Personally Identifiable Information, PHI: Protected Health Information).

### No-code/low-code platforms to accelerate clinician-led DX

Traditionally, developing custom applications with generative AI has required specialized AI engineering teams, creating a potential gap between the resulting solution and the actual needs on the clinical front lines. No-code and low-code development platforms are key to overcoming this bottleneck and accelerating clinician-led digital transformation (DX).

These platforms provide an environment where AI applications can be built through intuitive graphical user interfaces (GUIs) with little to no programming. Tools such as Langflow (https://www.langflow.org/), Flowise(https://flowiseai.com/), and Dify (https://dify.ai/jp) are particularly well-suited for on-premises deployment. By integrating these visual builders with an on-premises, local LLM, it becomes possible to operate LLMs locally via a no-code/low-code interface, which is ideal for cost-effective experimentation and small-scale implementation, especially where data-handling restrictions apply. It should be noted, however, that organizations must design their own solutions for Service Level Agreements (SLAs) and audit logs.

Such GUI-based environments empower clinicians and researchers to, for example, create and deploy their own specialized chatbots that use the latest research papers on a specific disease as a knowledge source for RAG, enabling them to summarize and extract information conversationally. This means that healthcare professionals, who have the deepest understanding of clinical challenges, can rapidly prototype and implement solutions themselves, which is expected to significantly accelerate the cycle of process improvement.

In summary, ensuring reliability via RAG, guaranteeing security with on-premises LLMs, and democratizing development through no-code platforms are not merely independent strategies. Rather, their synergistic integration is key to building a sustainable ecosystem where clinicians can lead the rapid and safe development of trusted AI applications.

## Governance, ethics, and open challenges

While the benefits of generative AI in medicine and healthcare are substantial, its responsible implementation is imperative. The deployment of generative AI, in particular, is accompanied by numerous technical, ethical, and legal challenges that remain to be addressed. This Section organizes these key issues and discusses the necessity of a robust governance framework.

### Challenges originating from algorithms and data

The quality of a generative AI model's output is contingent upon its training data and algorithms, which presents a set of intrinsic challenges.

**Data bias and fairness**: AI models have a tendency to learn and amplify biases present in their training data. Due to historical and socioeconomic factors, medical data often contains skewed representations of certain races, genders, or geographic populations^27)^. This issue, which predates the advent of generative AI, has been reported in other domains; for example a recidivism risk prediction model in the U.S. was shown to have a higher false-positive rate for African Americans than for Caucasians, despite equivalent overall accuracy^28)^, and an online advertising delivery system that displayed more negative ads in response to searches for African American names^29)^. These cases illustrate that an AI trained on biased data carries the risk of delivering lower diagnostic accuracy or recommending inappropriate treatments for specific population groups. This problem is not limited to LLMs; for example, when using Diffusion Models for data augmentation in rare diseases, biases in the source dataset could lead to the overrepresentation of certain pathological features or a loss of diversity. For a mathematical analysis demonstrating how algorithmic attempts to mitigate bias for one group can inadvertently harm that same group^30)^.

**Hallucinations and reliability**: As mentioned in Section 3, the tendency of LLMs to generate information that is not based on fact—hallucinations—is one of the greatest concerns for their application in medicine. Although techniques like RAG can mitigate this risk, complete elimination is currently difficult. It is therefore essential to design systems with a human-in-the-loop, particularly for critical decision-making processes.

**The black box problem and interpretability**: The deep learning models that underpin generative AI are characterized by complex internal decision- making processes, making it difficult for humans to intuitively understand the rationale behind their conclusions. This "black-box" nature poses a significant challenge. In a clinical setting, physicians are responsible for explaining the basis of their diagnoses and treatment plans. If the reasoning of an AI is opaque, clinicians can neither blindly accept its recommendations nor reject them, as they may be valid. Furthermore, if a 3D model generated by NeRF or 3DGS were to realistically render an anatomically incorrect structure, identifying the source of this error within the model would remain a formidable challenge.

### Legal and ethical issues specific to medicine

While the issues discussed in section 5.1 are universal, the introduction of generative AI into the medical domain gives rise to even more complex challenges.

**Patient privacy and data governance**: Although on-premises local LLMs (Section 4.2) are a powerful approach for ensuring data security, they are not sufficient on their own. It is essential to establish a robust data governance framework that defines who can access what data, for what purpose, and within what scope, even for internal data use.

**Accountability and the legal framework (EU-U.S.-Japan)**: When an AI-assisted clinical decision contributes to patient harm, accountability depends on how a jurisdiction defines (i) the responsible actors across the lifecycle and (ii) the binding obligations attached to each role.

In the EU, the AI Act allocates obligations by operator role (notably “provider” and “deployer”). For high-risk AI systems, providers must implement a quality management system, prepare and maintain technical documentation, ensure automatic record-keeping/logging, and establish a post-market monitoring system. Where an AI system qualifies as medical-device software/AI under the MDR/IVDR, those sectoral requirements apply in parallel. The MDCG 2025-6 FAQ (June 2025) provides non-binding practical guidance on the AI Act-MDR/IVDR interface, including when medical-device AI is high-risk under Article 6(1) of AI Act and how this relates to MDR/IVDR classification and conformity assessment pathways.

In the United States, the FDA oversees safety and effectiveness for AI-enabled medical devices within its jurisdiction, and FDA guidance has operationalized update pathways via PCCPs to support iterative model changes while maintaining reasonable assurance of safety and effectiveness. Downstream liability is typically handled under general tort and medical-malpractice doctrines rather than a unified federal AI-liability statute.

In Japan, accountability for medical AI is shaped by (a) regulation of AI that qualifies as a medical device (including SaMD) under the PMD Act, including post-market safety and change-control- related requirements; (b) operational governance and outsourcing controls under the MHLW *Guidelines for Safety Management of Medical Information Systems* (v6.0), which require documented “responsibility demarcation” between healthcare providers and vendors, including multi-vendor/cloud outsourcing chains; and (c) data-handling obligations under the APPI. A Japan-specific nuance is that the Product Liability Act defines a “product” as a “movable which is manufactured or processed,” which may limit strict-liability theories for standalone software (as opposed to devices embedding software), in contrast to the EU’s modernized product-liability framework that explicitly covers software/AI and certain update-related defects. In practice, this places greater emphasis on ex ante governance—documentation, auditability, logging, change control, and incident response—and on clear allocation of responsibilities among hospitals, clinicians, and vendors.

Furthermore, guidelines for the reporting and quality assessment of AI research are being developed. For example, the TRIPOD+AI^31)^ statement provides a checklist of items to include in manuscripts and abstracts to reduce the "black box" nature of AI models as much as possible. Similarly, PROBAST+AI^32)^ offers a standardized tool for assessing the quality of AI-based research, evaluating nearly 50 criteria related to the quality of model development, risk of bias, fairness, and the applicability of the AI to real-world problems.

Addressing these ethical and legal challenges, alongside establishing the evaluation and reporting guidelines outlined in this Section, is the essential foundation upon which generative AI can earn the trust of both clinicians and patients and be responsibly integrated into society.

## Discussion and future perspectives

In this paper, we have reviewed the impact of foundational generative AI technologies—the Transformer, the Diffusion Model, NeRF and 3DGS—across a wide range of applications in medicine and healthcare, from optimizing clinical workflows to advancing drug discovery and medical education. The automation of medical documentation by LLMs can significantly reduce the administrative burden on healthcare professionals, enabling more patient- centered care. Diffusion Models are contributing to improvements in the quality of diagnostic imaging and accelerating the drug discovery process, as exemplified by AlphaFold3, while NeRF and 3DGS promise to drive significant progress in surgical simulation and medical education.

However, as many of these technologies are still at the proof-of-concept stage, several critical challenges must be overcome before they can be implemented as safe and effective clinical tools. As discussed in this paper, the risk of hallucinations can be mitigated by RAG but is difficult to eliminate completely, suggesting that a human-in-the-loop operational model, wherein healthcare professionals retain final decision-making authority, is indispensable, particularly for life-critical decisions. Furthermore, while deploying local LLMs in an on-premises environment is a robust measure for ensuring data security when handling sensitive patient information, it is not sufficient on its own; this measure must be complemented by a rigorous data governance framework that strictly defines internal access privileges. Moreover, a societal consensus is urgently needed to address legal and ethical challenges, including the opacity of AI decision-making (the “black box problem”), inherent data biases, and the allocation of liability in cases of medical malpractice involving AI-assisted judgments.

Despite these challenges, generative AI has become indispensable for improving the quality of care. To realize its full potential, it is essential to establish an environment where healthcare professionals—who best understand the needs of the clinical front line—can rapidly and safely customize and experiment with AI, for instance through no- code/low-code development platforms. This must be done in parallel with the development of guidelines and legal frameworks to ensure the technology's safety and efficacy. Doing so will build the foundation of trust from both clinicians and patients, creating a future where AI does not replace but rather augments the capabilities of healthcare professionals.

## Author contributions

The first draft was written by TN and first revised by WU. All authors commented on and contributed to subsequent revisions, and all approved the final manuscript.

## Conflicts of interest statement

The authors declare no commercial or financial relationships that could be construed as a potential conflict of interest.
